# Supplementary material for: Improving Balance and Movement Control in Fencing Using IoT and Real-Time Sensorial Feedback
Source: Sensors (Basel). 2023 Dec 13;23(24):9801. doi: 10.3390/s23249801 (PMC10747936; doi:10.3390/s23249801)
Supplement: Supplementary file 1 [file sensors-23-09801-s001.zip › sensors-2688249-supplementary.pdf]

Table S1. Week 0 measurements of the control group

Subject	Total movement time [s]	Unbalance time [%] 2/3 axis	Unbalance time [%] 3/3 axis
CG1	11.04	41.17	24.51
CG2	11.9	42.61	17.39
CG3	10.1	23.47	13.26
CG4	8.14	18.99	7.59
CG5	11.03	50.47	23.36
CG6	9.89	29.16	12.5
CG7	8.39	49.38	28.39
CG8	9.84	38.94	17.89
CG9	9.89	31.25	10.41
CG10	9.3	18.22	16.22
**Average**	**9.952**	**34.366**	**17.152**

Table S2. Week 0 measurements of the test group with visual feedback

Subject	Total movement time [s]	Unbalance time [%] 2/3 axis	Unbalance time [%] 3/3 axis
VG1	10.24	31.37	12.23
VG2	10.17	37.61	18.26
VG3	12.17	18.92	9.11
VG4	9.42	42.83	24.22
VG5	10.21	46.37	20.22
VG6	10.49	27.46	15.89
VG7	11.31	28.78	12.33
VG8	10.63	39.24	27.37
VG9	11.09	35.15	18.29
VG10	10.88	22.32	13.49
**Average**	**10.661**	**33.005**	**17.141**

Table S3. Week 0 measurements of the test group with haptic feedback

Subject	Total movement time [s]	Unbalance time [%] 2/3 axis	Unbalance time [%] 3/3 axis
HG1	13.25	29.92	14.91
HG2	13.64	22.17	12.42
HG3	10.08	34.35	18.22
HG4	11.77	19.84	7.45
HG5	9.08	43.57	27.31
HG6	10.95	26.22	15.22
HG7	12.56	37.91	21.71
HG8	9.94	25.93	14.21
HG9	8.05	39.32	19.22
HG10	10.01	31.05	15.13
**Average**	**10.933**	**31.028**	**16.58**

Table S4. Week 5 measurements of the control group

Subject	Total movement time [s]	Unbalance time [%] 2/3 axis	Unbalance time [%] 3/3 axis
CG1	10.54	39.28	27.42
CG2	10.99	36.97	16.19
CG3	10.31	25.14	12.99
CG4	9.42	16.88	8.72
CG5	10.62	45.12	21.09
CG6	9.61	33.67	11.75
CG7	8.75	53.51	31.24
CG8	10.21	32.74	14.58
CG9	9.41	27.82	9.86
CG10	9.42	16.62	15.21
**Average**		**32.775**	**16.905**

Table S5. Week 5 measurements of the test group with visual feedback

Subject	Total movement time [s]	Unbalance time [%] 2/3 axis	Unbalance time [%] 3/3 axis
VG1	10.54	25.87	13.45
VG2	10.26	40.31	14.78
VG3	12.02	18.82	7.75
VG4	11.88	31.43	16.37
VG5	10.37	34.18	14.35
VG6	10.31	22.26	18.92
VG7	11.47	26.12	10.46
VG8	10.96	30.89	17.87
VG9	11.14	26.91	13.93
VG10	10.56	22.12	10.79
**Average**	**10.951**	**27.891**	**13.867**

Table S6. Week 5 measurements of the test group with haptic feedback

Subject	Total movement time [s]	Unbalance time [%] 2/3 axis	Unbalance time [%] 3/3 axis
HG1	12.87	27.41	12.83
HG2	12.96	23.66	15.74
HG3	9.96	33.95	16.86
HG4	10.78	21.74	10.21
HG5	9.39	36.37	20.41
HG6	11.06	22.68	17.89
HG7	12.24	35.41	18.41
HG8	10.27	27.18	14.61
HG9	9.37	36.91	16.38
HG10	10.58	32.85	14.32
**Average**	**10.948**	**29.816**	**15.766**

Table S7. The Wilcoxon test was applied to the control group based on unbalanced time on 2 out of 3 axes.

Subject	W0 - 2/3	W5 - 2/3	Diff.	Sign	Abs. diff.	Rank	Rank with sign		
CG1	41.17	39.28	1.89	1	1.89	3	3	The sum of ranks of negative diff.	15
CG2	42.61	36.97	5.64	1	5.64	9	9	The sum of ranks of negative diff.	37
CG3	23.47	25.14	-1.67	-1	1.67	2	-2		
CG4	18.99	16.88	2.11	1	2.11	4	4	Wilcoxon test result	15
CG5	50.47	45.12	5.35	1	5.35	8	8	Critical value	8
CG6	29.16	33.67	-4.51	-1	4.51	7	-7	Significance level	0.05
CG7	49.38	53.51	-4.13	-1	4.13	6	-6		
CG8	38.94	32.74	6.2	1	6.2	10	10		
CG9	31.25	27.82	3.43	1	3.43	5	5		
CG10	18.22	16.62	1.6	1	1.6	1	1		

Table S8. The Wilcoxon test was applied to the control group based on unbalanced time on 3 out of 3 axes.

Subject	W0 - 3/3	W5 - 3/3	Diff.	Sign	Abs. diff.	Rank	Rank with sign		
CG1	24.51	27.42	-2.91	-1	2.91	9	-9	The sum of ranks of negative diff.	22
CG2	17.39	16.19	1.2	1	1.2	6	6	The sum of ranks of negative diff.	33
CG3	13.26	12.99	0.27	1	0.27	1	1		
CG4	7.59	8.72	-1.13	-1	1.13	5	-5	Wilcoxon test result	22
CG5	23.36	21.09	2.27	1	2.27	7	7	Critical value	8
CG6	12.5	11.75	0.75	1	0.75	3	3	Significance level	0.05
CG7	28.39	31.24	-2.85	-1	2.85	8	-8		
CG8	17.89	14.58	3.31	1	3.31	10	10		
CG9	10.41	9.86	0.55	1	0.55	2	2		
CG10	16.22	15.21	1.01	1	1.01	4	4		

Table S9. The Wilcoxon test was applied to the group training with visual feedback based on unbalanced time on 2 out of 3 axes.

Subject	W0 - 2/3	W5 - 2/3	Diff.	Sign	Abs. diff.	Rank	Rank with sign		
VG1	31.37	25.87	5.5	1	5.5	6	6	The sum of ranks of negative diff.	4
VG2	37.61	40.31	-2.7	-1	2.7	4	-4	The sum of ranks of negative diff.	45
VG3	18.92	18.82	0.1	1	0.1	1	1		
VG4	42.83	31.43	11.4	1	11.4	9	9	Wilcoxon test result	4
VG5	46.37	34.18	12.19	1	12.19	10	10	Critical value	8
VG6	27.46	22.26	5.2	1	5.2	5	5	Significance level	0.05
VG7	28.78	26.12	2.66	1	2.66	3	3		
VG8	39.24	30.89	8.35	1	8.35	8	8		
VG9	35.15	26.91	8.24	1	8.24	7	7		
VG10	22.32	22.12	0.2	1	0.2	2	2		

Table S10. The Wilcoxon test was applied to the group training with visual feedback based on unbalanced time on 3 out of 3 axes.

Subject	W0 - 3/3	W5 - 3/3	Diff.	Sign	Abs. diff.	Rank	Rank with sign		
VG1	13.45	12.23	1.22	1	1.22	2	2	The sum of ranks of negative diff.	47
VG2	14.78	18.26	-3.48	-1	3.48	6	-6	The sum of ranks of negative diff.	6
VG3	7.75	9.11	-1.36	-1	1.36	3	-3		
VG4	16.37	24.22	-7.85	-1	7.85	9	-9	Wilcoxon test result	6
VG5	14.35	20.22	-5.87	-1	5.87	8	-8	Critical value	8
VG6	18.92	15.89	3.03	1	3.03	5	5	Significance level	0.05
VG7	10.46	10.33	0.13	1	0.13	1	1		
VG8	17.87	27.37	-9.5	-1	9.5	10	-10		
VG9	13.93	18.29	-4.36	-1	4.36	7	-7		
VG10	10.79	13.49	-2.7	-1	2.7	4	-4		

Table S11. The Wilcoxon test was applied to the group training with visual feedback based on unbalanced time on 2 out of 3 axes.

Subject	W0 - 2/3	W5 - 2/3	Diff.	Sign	Abs. diff.	Rank	Rank with sign		
HG1	29.92	27.41	2.51	1	2.51	8	8	The sum of ranks of negative diff.	14
HG2	22.17	23.66	-1.49	-1	1.49	3	-3	The sum of ranks of negative diff.	33
HG3	34.35	33.95	0.4	1	0.4	1	1		
HG4	19.84	21.74	-1.9	-1	1.9	5	-5	Wilcoxon test result	14
HG5	43.57	36.37	7.2	1	7.2	10	10	Critical value	8
HG6	26.22	22.68	3.54	1	3.54	9	9	Significance level	0.05
HG7	37.91	35.41	2.5	1	2.5	7	7		
HG8	25.93	27.18	-1.25	-1	1.25	2	-2		
HG9	39.32	36.91	2.41	1	2.41	6	6		
HG10	31.05	32.85	-1.8	-1	1.8	4	-4		

Table S12. The Wilcoxon test was applied to the group training with visual feedback based on unbalanced time on 3 out of 3 axes.

Subject	W0 - 3/3	W5 - 3/3	Diff.	Sign	Abs. diff.	Rank	Rank with sign		
HG1	12.83	14.91	-2.08	-1	2.08	4	-4	The sum of ranks of negative diff.	34
HG2	15.74	12.42	3.32	1	3.32	9	9	The sum of ranks of negative diff.	21
HG3	16.86	18.22	-1.36	-1	1.36	3	-3		
HG4	10.21	7.45	2.76	1	2.76	6	6	Wilcoxon test result	21
HG5	20.41	27.31	-6.9	-1	6.9	10	-10	Critical value	8
HG6	17.89	15.22	2.67	1	2.67	5	5	Significance level	0.05
HG7	18.41	21.71	-3.3	-1	3.3	8	-8		
HG8	14.61	14.21	0.4	1	0.4	1	1		
HG9	16.38	19.22	-2.84	-1	2.84	7	-7		
HG10	14.32	15.13	-0.81	-1	0.81	2	-2		
